# Supplementary material for: IFN-γ primes bone marrow neutrophils to acquire regulatory functions in severe viral respiratory infections
Source: Sci Adv. 2024 Oct 11;10(41):eadn3257. doi: 10.1126/sciadv.adn3257 (PMC11468905; doi:10.1126/sciadv.adn3257)
Supplement: Supplementary file 1 — Figs. S1 to S9 Legend for table S1 Table S2 [file sciadv.adn3257_sm.pdf]

Science Advances
AAAS

Supplementary Materials for

IFN-γ primes bone marrow neutrophils to acquire regulatory functions in severe viral respiratory infections

Florent Creusat *et al.*

Corresponding author: Christophe Paget, christophe.paget@inserm.fr

Sci. Adv. **10**, eadn3257 (2024)
DOI: 10.1126/sciadv.adn3257

The PDF file includes:

Figs. S1 to S9
Legend for table S1
Table S2

Other Supplementary Material for this manuscript includes the following:

Table S1

Supplementary Figures

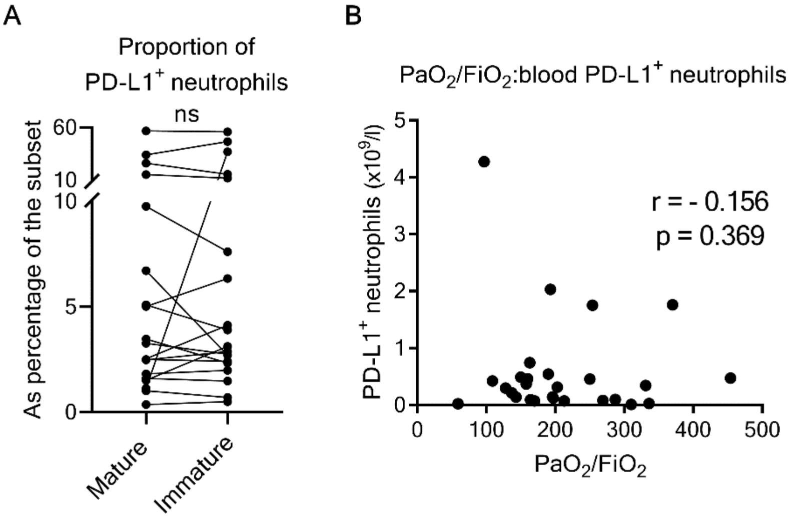

Figure S1: PD-L1+ neutrophils in blood of patients with severe VRI. A, Proportion of blood PD-L1+ neutrophils in mature vs immature subsets. **B**, Spearman's rank correlation of blood PD-L1+ neutrophils and hypoxemia levels on admission of intubated patients with viral pneumonia (n =27). Ns, not significant.

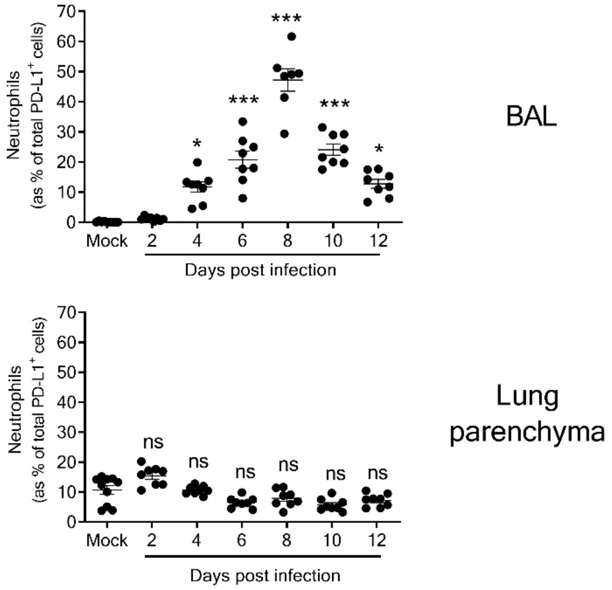

Figure S2: Frequency of neutrophils within total PD-L1+ leukocytes. Relative proportion of neutrophils within total PD-L1+ leukocytes in airways and lung parenchyma was evaluated by flow cytometry. Individual and means ± SEM pooled from three independent experiments are shown in the right panel (8-10 mice/group). ns, not significant; *, $p < 0.05$; ***, $p < 0.001$.

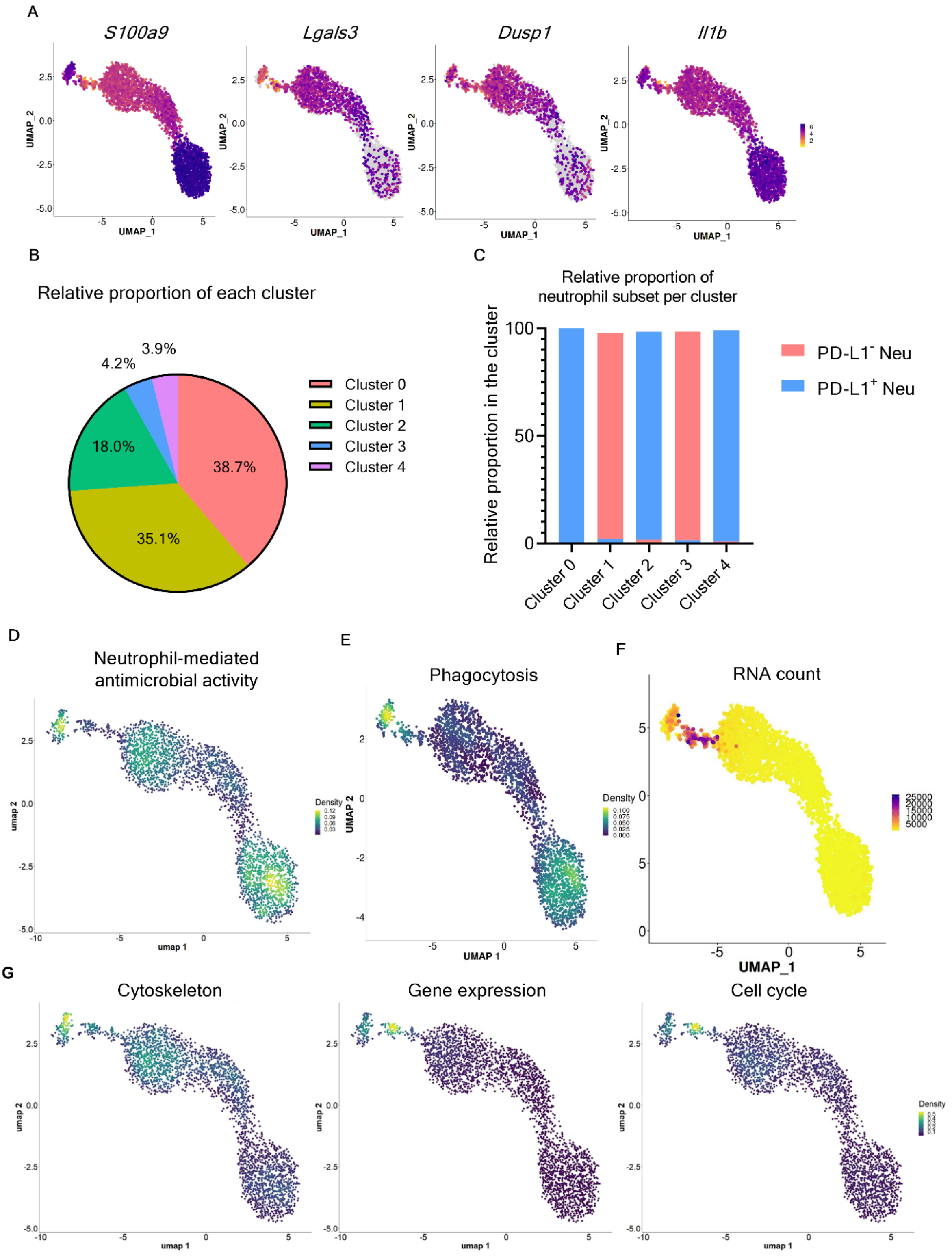

Figure S3: scRNA-seq of lung neutrophilic subsets based on PD-L1 expression in IAV-infected C57Bl/6j mice. A, Expression map of selected neutrophil gene markers. **B**, Relative proportion of each cluster in merged data set. **C**, Relative proportion of PD-L1^{-} and PD-L1^{+} subsets in each defined cluster. **D-E**, Neutrophil-mediated antimicrobial activity (**D**) and phagocytosis (**E**) signatures along merged data. **F**, Number of reads per cell along merged data **G**, Expression of signature from cytoskeleton, gene expression (nFeatures) and cell cycle.

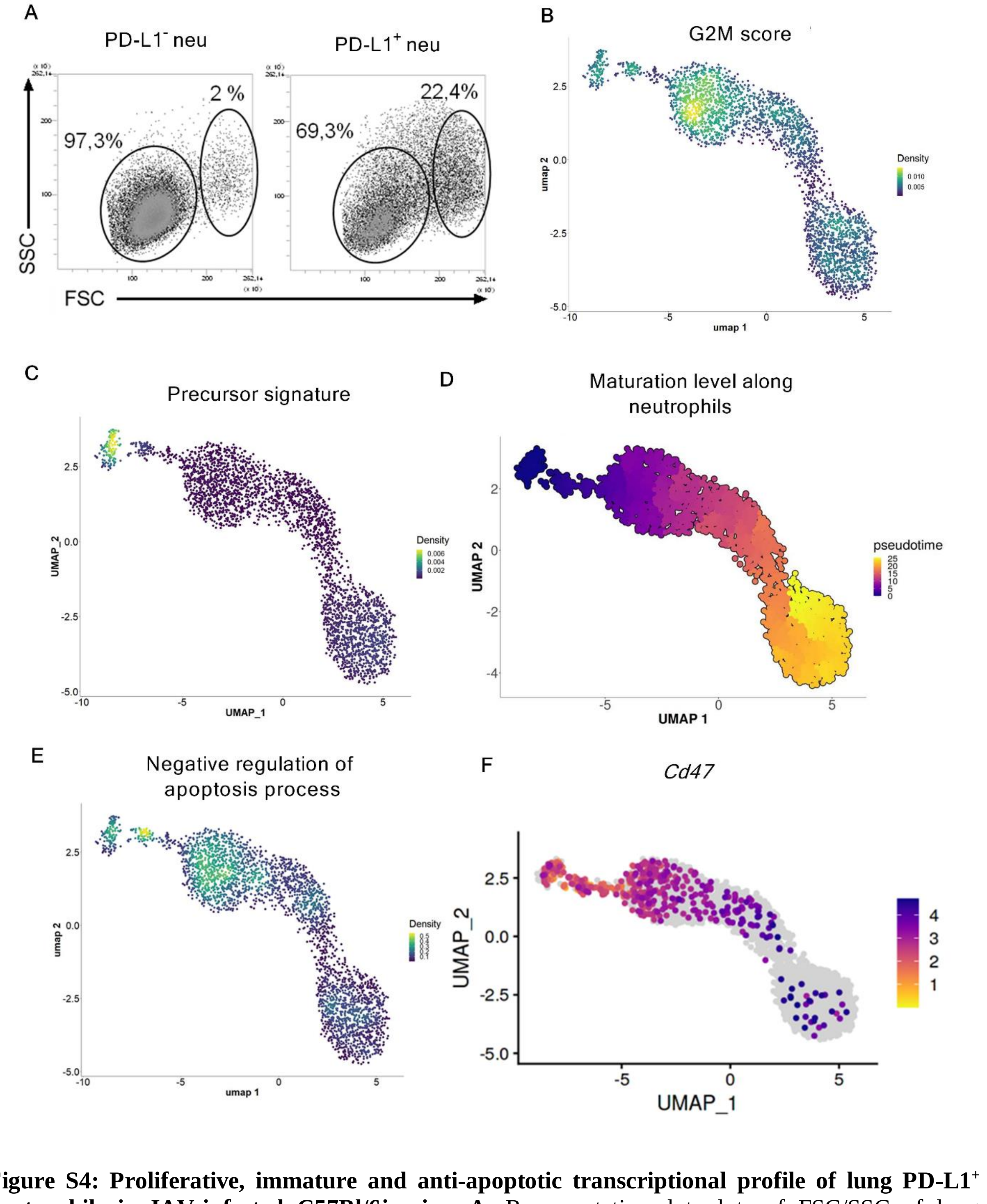

Figure S4: Proliferative, immature and anti-apoptotic transcriptional profile of lung PD-L1⁺ neutrophils in IAV-infected C57Bl/6j mice. **A,** Representative dot plots of FSC/SSC of lung neutrophil subsets from IAV-infected mice. **B,** Density plot of G2M cell cycle score were extracted from Seurat package. **C**, Density plot of the "precursor" signature in neutrophil transcriptomes. **D**, Maturation level mapped with pseudotime along merged data with precursor signature used as root. **E**, "Negative regulation of apoptosis process" signature in neutrophil transcriptomes. **F**, Expression map of *Cd47* in neutrophil transcriptomes

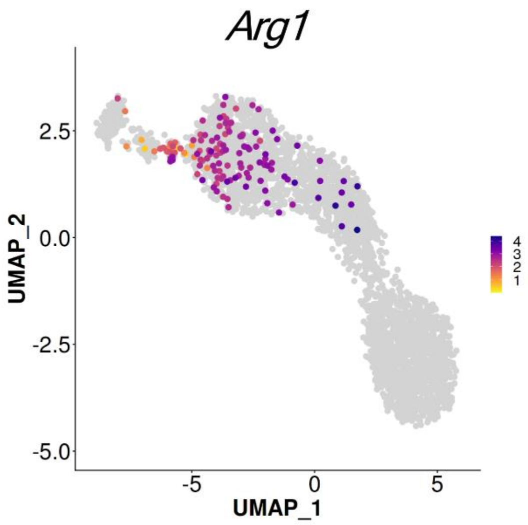

Figure S5: Lung PD-L1⁺ neutrophils from IAV-infected C57Bl/6j mice expressed *Arg1*. Expression map of *Arg1* in neutrophil transcriptomes from **Fig. 3**.

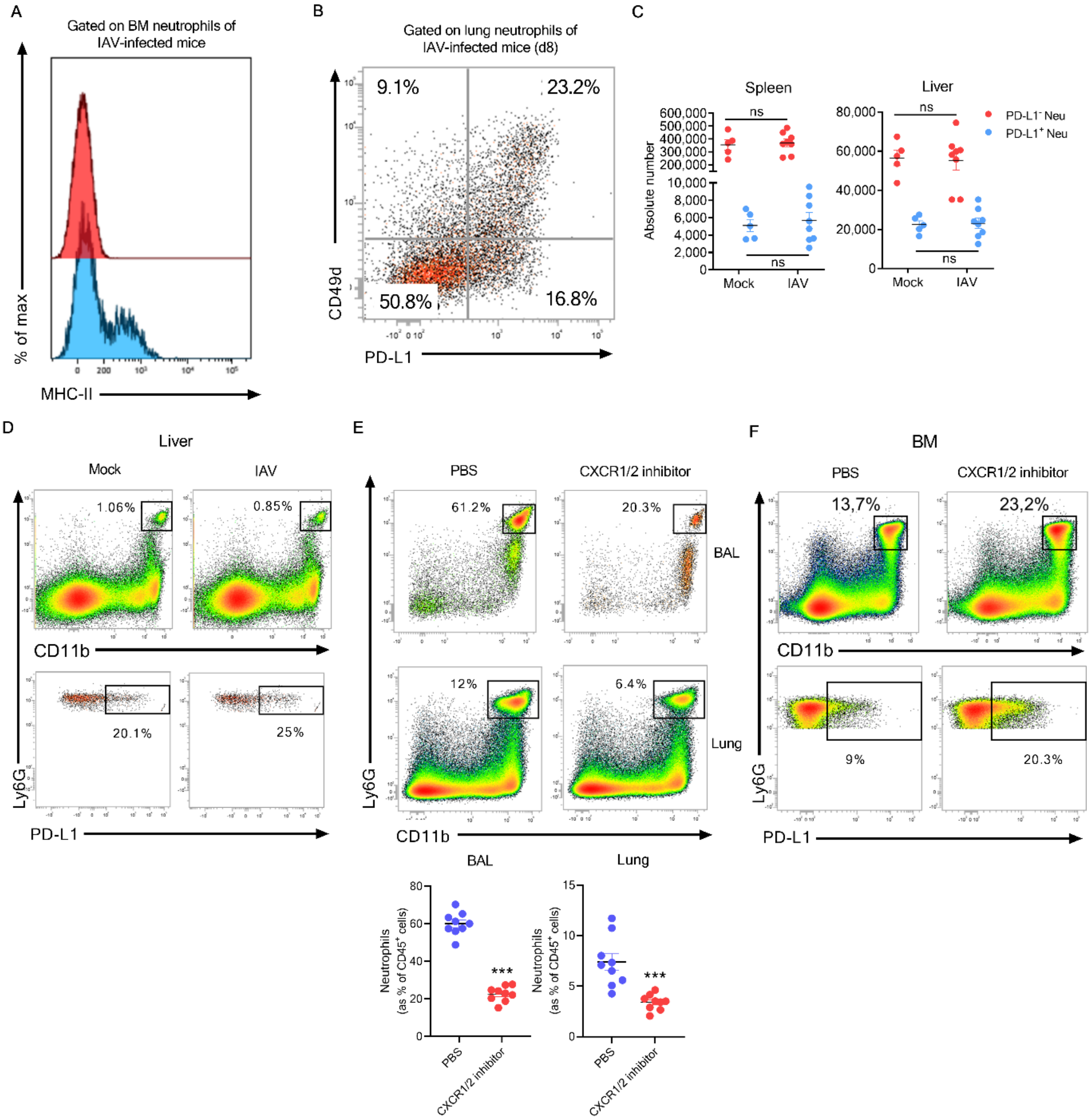

Figure S6: Origin and migration of IAV-induced PD-L1$^+$ neutrophils in C57Bl/6j mice. A, Flow cytometry expression of MHC-II on BM neutrophil subsets. Representative dot plots of 4 independent experiments of PD-L1$^-$ and PD-L1$^+$ BM neutrophils from mock or IAV-infected mice are shown. **B**, Flow cytometry expression of CD49d on lung neutrophils from IAV-infected mice according to PD-L1 expression. Representative dot plots of 3 independent experiments are shown. **C**, Absolute number of neutrophil subsets in spleen and liver of mock and IAV-infected mice. Individual values and means ± SEM from two independent experiments are shown (n = 5-8/group). **D**, Proportion of PD-L1$^+$ neutrophils in the liver of mock and IAV-infected mice. Representative dot plots of 3 independent experiments are shown. **E-F**, Proportion of neutrophils subsets in IAV-infected mice treated or not with reparixin (CXCR1/2 inhibitor). **E**, Neutrophil proportions in BAL and lung parenchyma of IAV-infected mice treated or not with reparixin. Representative dot plots from two independent experiments are shown in the upper panel. Individual values and means ± SEM from two independent experiments are shown (n = 9/group) in the lower panel. **F**, Neutrophil proportions in BM of IAV-infected mice treated or not with reparixin. Representative dot plots from two independent experiments are shown. ns, not significant; ***, $p < 0.001$.

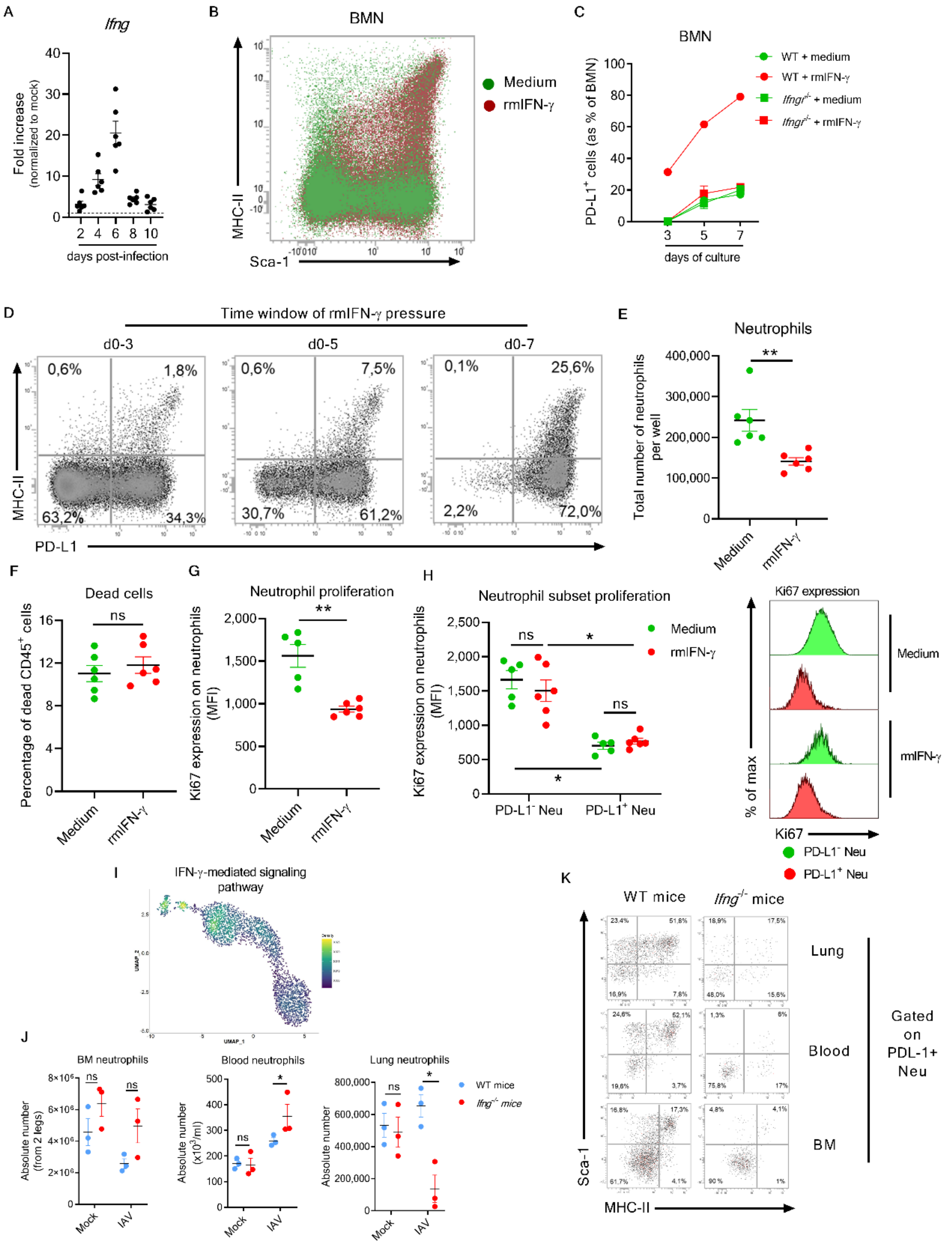

Figure S7: Influence of IFN-γ on emergence of BM PD-L1⁺ neutrophils. A, *Ifng* mRNA expression in BM was determined by RT-qPCR. Data are normalized to expression of *Gapdh* and are expressed as fold increase over average gene expression in mock-treated mice. Individual values and means ± SEM from two independent experiments are shown (n = 6/group). **B**, Flow cytometry expression of Sca-1 and MHC-II on BMN differentiated in presence or not of rmIFN-γ. Representative overlay dot plots are shown. **C**, Kinetic representation of the proportion of PD-L1$^+$ BMN differentiated from WT or *Ifngr*$^{-/-}$ BM stem cells in presence or not of rm-IFN-γ. Means ± SEM from two independent experiments are shown. **D**, Representative dot plots showing PD-L1 and MHC-II expression on differentiated BMN according to the length of rm-IFN-γ are shown. **E**, Effect of rm-IFN-γ on the absolute number of BMN obtained at the end of the differentiation protocol. Individual values and means ± SEM from two independent experiments are shown. **F**, Relative proportion of dead cells at the end of the protocol

defined using the Live/Dead stain kit. Individual values and means ± SEM from two independent experiments are shown. **G**, Flow cytometry expression of the proliferation marker Ki67 in differentiated BMN in presence or not rm-IFN-γ. Individual values and means ± SEM from two independent experiments are shown. **H**, Differential proliferation rate in PD-L1$^-$ vs PD-L1$^+$ BMN measured by Ki67 expression. Individual values and means ± SEM from two independent experiments are shown. Representative overlay histograms are shown in the right panel. **I**, IFN-γ-mediated signalling pathway signature in neutrophil transcriptomes. **J**, Absolute numbers of neutrophils in BM, blood and lung of IAV-infected WT or *Ifng*$^{-/-}$ mice. **K**, Representative dot plots of one experiment out of two of Sca-1 and MHC-II expression in lung, blood and BM PD-L1$^+$ neutrophils from IAV-infected WT or *Ifng*$^{-/-}$ mice. ns, not significant; *, $p < 0.05$; **, $p < 0.01$.

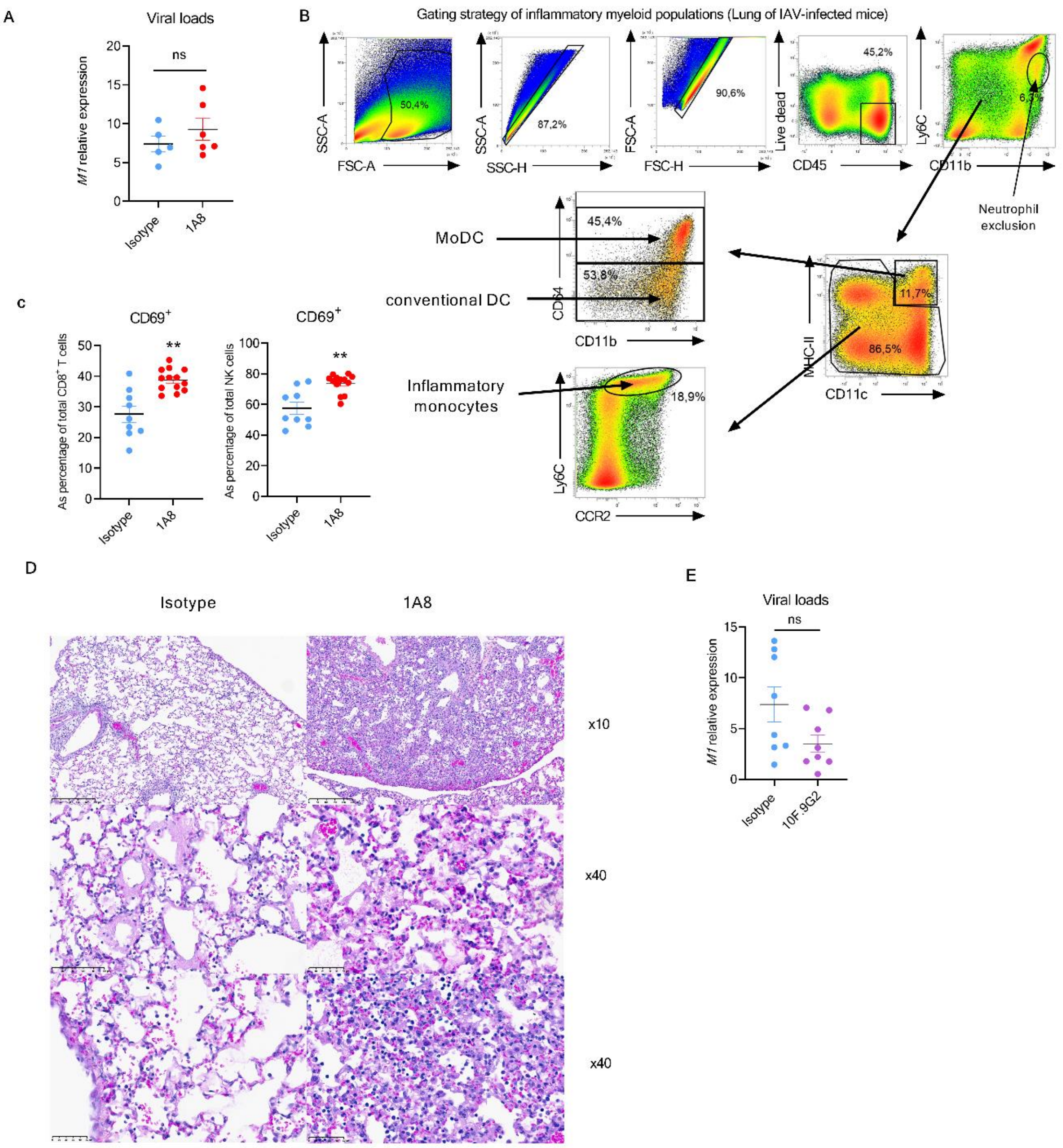

Figure S8: Effect of neutrophil depletion and/or PD-L1 blockade on viral loads and IAV-induced immunopathology. A-D, Mice were treated from 4 dpi and every second day with isotype control or 1A8. **A**, Analysis of the viral loads in the lung of IAV-infected WT mice treated with Ig control or 1A8. IAV *M1* mRNA relative expression in the whole lung were measured by quantitative RT-PCR. Individual values and means ± SEM from two independent experiments are shown. **B**, Gating strategy of the lung inflammatory myeloid subsets. Representative dot plots of one experiment out of 6 are shown. **C-D**, Mice were euthanized on day 8 and lungs were collected. **C**, Individual values and means ± SEM of CD8^{+} T cells (CD45^{+} CD3^{+} CD8α^{+}) and NK cells (CD45^{+} CD3^{-} NK1.1^{+}) from two independent experiments are shown (9-13 mice/group). **D**, Comparative analysis of lung injury. In the top panels, representative lung sections indicative of higher perivascular and peribronchic infiltrates combined with severe acute lung injury in 1A8-treated mice are shown. Middle panels show increased fibrin debris and edema in 1A8-treated mice. Low panels are indicative of increased alveolar septal thickening and mononuclear cell infiltrates in 1A8-treated mice. **E**, Analysis of the viral load in the lung of IAV-infected WT mice treated with Ig control or 10F.9G2. IAV *M1* mRNA relative expression in the whole lung were measured by quantitative RT-PCR. Individual values and means ± SEM from two independent experiments are shown. ns, not significant.

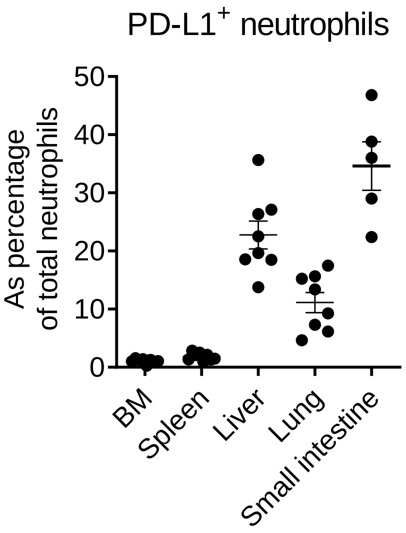

Figure S9: Tissue distribution of PD-L1+ neutrophils at homeostasis. Relative proportion of PD-L1+ neutrophils within total neutrophils in various peripheral tissues was evaluated by flow cytometry. Individual and means ± SEM pooled from at least two independent experiments are shown in the right panel (5-8 mice/group).

Table S1: List of differentially up-regulated genes per neutrophil cluster. This table can be found as a separate excel file.

Table S2: List of primers used in the study.

Gene	5' to 3'	Primers
Areg	Sense	GGTCTTAGGCTCAGGCCATTA
	Anti-Sense	CGCTTATGGTGGAAACCTCTC
Angpt1	Sense	CACATAGGGTGCAGCAACCA
	Anti-Sense	CGTCGTGTTCTGGAAGAATGA
Angpt2	Sense	CCTCGACTACGACGACTCAGT
	Anti-Sense	TCTGCACCACATTCTGTTGGA
Arg1	Sense	CTCCAAGCCAAAGTCCTTAGAG
	Anti-Sense	AGGAGCTGTCATTAGGGACATC
Ccl2	Sense	TTAAAAACCTGGATCGGAACCAA
	Anti-Sense	GCATTAGCTTCAGATTTACGGGT
Csf2	Sense	GGCCTTGGAAGCATGTAGAGG
	Anti-Sense	GGAGAACTCGTTAGAGACGACTT
Cxcl1	Sense	CTGGGATTCACCTCAAGAACATC
	Anti-Sense	CAGGGTCAAGGCAAGCCTC
Cxcr2	Sense	ATGCCCTCTATTCTGCCAGAT
	Anti-Sense	GTGCTCCGGTTGTATAAGATGAC
Dll4	Sense	TTCCAGGCAACCTTCTCCGA
	Anti-Sense	ACTGCCGCTATTCTTGTCCC
Furin	Sense	AGGGACGTGTATCAGGAGCC
	Anti-Sense	CCTGCTAGGTCGGGATGATTC

Gapdh	Sense	AGGTCGGTGTGAACGGATTTG
	Anti-Sense	TGTAGACCATGTAGTTGAGGTCA
Hmgb1	Sense	GGCGAGCATCCTGGCTTATC
	Anti-Sense	GGCTGCTTGTCATCTGCTG
Ifng	Sense	ATGAACGCTACACACTGCATC
	Anti-Sense	CCATCCTTTTGCCAGTTCCTC
Igf1	Sense	CTGGACCAGAGACCCTTTGC
	Anti-Sense	GGACGGGGACTTCTGAGTCTT
Il13	Sense	CCTGGCTCTTGCTTGCCTT
	Anti-Sense	GGTCTTGTGTGATGTTGCTCA
Il17a	Sense	TTTAACTCCCTTGGCGCAAAA
	Anti-Sense	CTTTCCCTCCGCATTGACAC
Il17rb	Sense	GGCTGCCTAAACCACGTAATG
	Anti-Sense	CCCGTTGAATGAGAATCGTGT
Il1b	Sense	GCAACTGTTCCTGAACTCAACT
	Anti-Sense	ATCTTTTGGGGTCCGTCAACT
Il22	Sense	ATGAGTTTTTCCCTTATGGGGAC
	Anti-Sense	GCTGGAAGTTGGACACCTCAA
Il6	Sense	TAGTCCTTCCTACCCCAATTTCC
	Anti-Sense	TTGGTCCTTAGCCACTCCTTC
Mmp10	Sense	GAGCCACTAGCCATCCTGG
	Anti-Sense	CTGAGCAAGATCCATGCTTGG
Mmp13	Sense	CTTCTTCTTGTTGAGCTGGACTC
	Anti-Sense	CTGTGGAGGTCACTGTAGACT
Mmp3	Sense	ACATGGAGACTTTGTCCCTTTTG
	Anti-Sense	TTGGCTGAGTGGTAGAGTCCC
Cd274	Sense	GCTCCAAAGGACTTGTACGTG
	Anti-Sense	TGATCTGAAGGGCAGCATTTC
Ptges2	Sense	CCTCGACTTCCACTCCCTG
	Anti-Sense	TGAGGGCACTAATGATGACAGAG
S100a8	Sense	AAATCACCATGCCCTCTACAAG
	Anti-Sense	CCCACTTTTATCACCATCGCAA
Smox	Sense	TCCCACGGGAATCCTATCTATC
	Anti-Sense	GCCACGGTTGGTAAGGTAGC
Tgfb1	Sense	CTCCCGTGGCTTCTAGTGC
	Anti-Sense	GCCTTAGTTTGGACAGGATCTG
Tnfa	Sense	CCCTCACACTCAGATCATCTTCT
	Anti-Sense	GCTACGACGTGGGCTACAG
Tnfaip3	Sense	GAACAGCGATCAGGCCAGG
	Anti-Sense	GGACAGTTGGGTGTCTCACATT
Vegfa	Sense	GCACATAGAGAGAATGAGCTTCC
	Anti-Sense	CTCCGCTCTGAACAAGGCT
